# The repertoire of equine intestinal α-defensins

**DOI:** 10.1186/1471-2164-10-631

**Published:** 2009-12-23

**Authors:** Oliver Bruhn, Sven Paul, Jens Tetens, Georg Thaller

**Affiliations:** 1 Institute of Animal Breeding and Husbandry, Christian-Albrechts-University of Kiel, Hermann-Rodewald-Straße 6, D-24118 Kiel, Germany

## Abstract

**Background:**

Defensins represent an important class of antimicrobial peptides. These effector molecules of the innate immune system act as endogenous antibiotics to protect the organism against infections with pathogenic microorganisms. Mammalian defensins are classified into three distinct sub-families (α-, β- and θ-defensins) according to their specific intramolecular disulfide-bond pattern. The peptides exhibit an antimicrobial activity against a broad spectrum of microorganisms including bacteria and fungi. Alpha-Defensins are primarily synthesised in neutrophils and intestinal Paneth cells. They play a role in the pathogenesis of intestinal diseases and may regulate the flora of the intestinal tract. An equine intestinal α-defensin (DEFA1), the first characterised in the *Laurasiatheria*, shows a broad antimicrobial spectrum against human and equine pathogens. Here we report a first investigation of the repertoire of equine intestinal α-defensins. The equine genome was screened for putative α-defensin genes by using known α-defensin sequences as matrices. Based on the obtained sequence information, a set of oligonucleotides specific to the α-defensin gene-family was designed. The products generated by reverse-transcriptase PCR with cDNA from the small intestine as template were sub-cloned and numerous clones were sequenced.

**Results:**

Thirty-eight equine intestinal α-defensin transcripts were determined. After translation it became evident that at least 20 of them may code for functional peptides. Ten transcripts lacked matching genomic sequences and for 14 α-defensin genes apparently present in the genome no appropriate transcript could be verified. In other cases the same genomic exons were found in different transcripts.

**Conclusions:**

The large repertoire of equine α-defensins found in this study points to a particular importance of these peptides regarding animal health and protection from infectious diseases. Moreover, these findings make the horse an excellent species to study biological properties of α-defensins. Interestingly, the peptides were not found in other species of the *Laurasiatheria *to date. Comparison of the obtained transcripts with the genomic sequences in the current assembly of the horse (EquCab2.0) indicates that it is yet not complete and/or to some extent falsely assembled.

## Background

Antimicrobial peptides are effector molecules of the innate immune system which provides the first line of defense against a wide variety of microbes [1]. These peptides act as endogenous antibiotics protecting the organism against infections with pathogenic microorganisms [2]. Antimicrobial peptides are synthesised by circulating phagocytic cells, leucocytes and epithelial cells of mucosal tissues. Defensins are an important class of antimicrobial peptides which can be found in plants [3], invertebrates [4] and vertebrates [5]. Defensins are cationic and cysteine-rich peptides with a molecular structure consisting of three antiparallel β-sheets [2]. They contain six highly conserved cysteine residues forming characteristic intramolecular disulfide bonds. Mammalian defensins are classified into three distinct sub-families due to the disulfide array: α-, β- und θ-defensins [6]. The peptides exhibit a direct antimicrobial activity against a broad spectrum of microorganisms including Gram-negative and Gram-positive bacteria [7], fungi [8] and enveloped viruses [9]. Defensins are thought to kill bacteria by an initial electrostatic interaction with the negatively charged phospholipids of the microbial cytoplasmatic membrane, followed by membrane permeabilisation and lysis of the microbes [10,11].

Unlike β-defensins that have been found in numerous tissues and in all mammals studied so far, α-defensins are presumably unique to a few tissues and are absent in some mammalian species. Alpha-defensin gene expression was observed in humans, mice [12], rhesus macaques [13], rats [14], rabbits [15], guinea pigs [16], hamsters [17] and the horse [18]. They were also identified *in silico *in the genome of the opossum [19], elephant, and hedgehog tenrec [20]. The peptides are absent in the genomes of cattle [21] and dog [22]. Additionally, α-defensins are mainly synthesised in neutrophilic granulocytes and in Paneth cells [23]. Paneth cells are secretory epithelial cells, which are most abundant in the distal small intestine at the base of the crypts of Lieberkuhn. Based on the current state of knowledge, the horse is the only species in the group of *Laurasiatheria *expressing α-defensins. The first equine α-defensin transcript was found in the intestine of the horse [18]. The authors showed that the transcript, named DEFA1, was exclusively produced in the small intestine together with another α-defensin (DEFA5L), known from an equine BAC-clone [24]. Enteric α-defensins play an important role in the defence of the intestinal tract against microbes and may regulate the flora of enteric bacteria [25]. Dysfunctions in the regulation of intestinal α-defensins or mutations can lead to serious diseases like Morbus Crohn [26], and to a higher susceptibility to inflammatory bowel diseases [27] or diarrhea [28].

The antimicrobial properties of recombinant equine DEFA1 were comprehensively studied. An antimicrobial effect was observed against 22 Gram-positive and Gram-negative bacterial strains and one yeast [18,29]. Among them are prominent human and equine pathogens like *Staphlylococcus aureus*, *Pseudomonas aeruginosa*, *Escherichia coli*, *Salmonella typhimurium*, *Streptococcus equi *and *Rhodococcus equi*.

The antimicrobial potency of equine DEFA1 and the general importance of intestinal defensins on animal health make them interesting candidates for further antimicrobial and functional characterisations. This motivated us to analyse the repertoire of the equine intestinal α-defensins in detail. Initial genomic *in silico *analyses with DEFA1- and DEFA5L sequences as matrices led to an identification of numerous unknown putative α-defensin sequences. Based on this information a set of oligonucleotides specific to the α-defensin gene-family was designed and RT-PCR products were generated with cDNA from the small intestine as template.

## Methods

### Tissue collection and RNA extraction

Tissue from the centre of the outstreched small intestine of one horse was collected immediately after death during slaughtering and was frozen in liquid nitrogen. Approval of an ethics committee was not necessary since the tissue collection was done during the routine processes in an abattoir. Approximately 300 mg of frozen tissue were disrupted with a mortar and homogenised using a rotor-stator homogeniser. Total RNA was extracted by using the RNeasy Midi Kit (Qiagen, Hilden, Germany) according to the manufacturer's guidelines. The RNA concentration was determined photometrically by measuring the UV-absorption of the sample. One unit of the optical density at 260 nm equates to a concentration of 40 μg total RNA. The extracted RNA was stored at -80°C.

### Genomic analysis with α-defensin sequences as matrices

By using the known equine α-defensin sequences DEFA5L ([GenBank:AM039964]; [24]) and DEFA1 ([GenBank:EF379126]; [18]) as matrices, a BLAST-search ("blastn") in the nr/nt- and wgs-databases of the horse assembly EquCab1.0 was performed to obtain nucleotide sequences resembling α-defensin genes. This genomic sequence information represented the basis to design a set of oligonucleotides for the following RT-PCR studies.

### Conceptual design of oligonucleotides for RT-PCR-reactions

Forward oligonucleotides were designed according to the genomic sequences determined by the *in silico *approach with following assumptions: (1) Oligonucleotides should include the ATG-start codon of the putative α-defensin genes or alternatively should bind a few bases upstream of the ATG so that in both cases the complete cDNA sequence could be amplified. (2) The set of forward oligonucleotides should amplify all the sequences determined by the *in silico *approach if combined with an oligo-dT-nucleotide used as a reverse oligonucleotide. Therefore some forward oligonucleotides contain wobble-bases. (3) Each forward oligonucleotide should amplify a single product in combination with the oligo-dT-nucleotide.

The 18 forward oligonucleotides and the oligo-dT-nucleotide are listed in Table 1. The oligonucleotides were synthesised by biomers.net (Ulm, Germany).

**Table 1:** Oligonucleotides for reverse-transcriptase PCR and sequencing reactions.

Name	Sequence	Target
alpha1	5'-gtgactsacggccatgaggac-3'	putative α-defensin
alpha2	5'-gtgactsacagccatgaggac-3'	putative α-defensin
alpha3	5'-gtgactsatatccatgaggac-3'	putative α-defensin
alpha4	5'-gtgactsatatacatgaggac-3'	putative α-defensin
alpha5	5'-atgactcacagccatgaagac-3'	putative α-defensin
alpha6	5'-gtgacccacagccatgaggac-3'	putative α-defensin
alpha7	5'-gtgactgacagccataaggac-3'	putative α-defensin
alpha2-1	5'-cctgacctccaggtgacccac-3'	putative α-defensin
alpha2-2	5'-cctgacctccagttgactccc-3'	putative α-defensin
alpha2-3	5'-attcacttccaggtgactcac-3'	putative α-defensin
alpha2-4	5'-ctggaactccaggtgactcac-3'	putative α-defensin
alpha2-5	5'-tctgaactccaggtgactcac-3'	putative α-defensin
alpha2-6	5'-ggcctcagtcaggtgactgac-3'	putative α-defensin
alpha2-7	5'-cccgacctctacatgactcac-3'	putative α-defensin
alpha2-8	5'-cctgacctccagatgactgac-3'	putative α-defensin
alpha2-9	5'-cctgaactccaggtgactsac-3'	putative α-defensin
alpha2-10	5'-cctgagctccaggtgactyac-3'	putative α-defensin
alpha2-11	5'-cctgagctccaggtgactccc-3'	putative α-defensin
Oligo-dT-Bio	5'-actctatgagaattcgatgagcgatctgt_(25)_v-3'	poly-A-tail
Tail-Primer3'	5'-actctatgagaattcgatgagcgatctg-3'	Oligo-dT-Bio
T7 promoter-oligo	5'-gtaatacgactcactatag-3'	pDrive cloning vector
SP6 promoter-oligo	5'-catttaggtgacactatag-3'	pDrive cloning vector

### RT-PCR

The cDNA was synthesised by using the Superscript-Transcriptase (Invitrogen, Carlsbad, USA) in combination with an oligo-dT-nucleotide (Oligo-dT-Bio, Table 1) according to Schramm et al. [30]. Total RNA from the small intestine served as a template. The cDNA was stored at -20°C.

PCR reactions were performed by using the forward oligonucleotides (Table 1) paired with the reverse primer (Tail-Primer3', Table 1) and the cDNA from the small intestine as template. The optimal annealing temperature for the PCR reactions was determined by performing a temperature-gradient-PCR in a range from 50-68°C. The reaction mixtures were incubated at 94°C for 3 min, followed by 33 cycles at 55°C (alpha1-alpha7), 58°C (alpha2-2, 2-6, 2-8), 60°C (alpha2-7), 62°C (alpha2-3, 2-9, 2-11) or 67°C (alpha2-1, 2-4, 2-5, 2-10) for 50 s, 68°C for 45 s, and 94°C for 30 s. A final extension step was performed at 68°C for 10 min. Hot-Start-polymerase (Eppendorf, Hamburg, Germany) was used for PCR-reactions (1.5 U/reaction) with the appropriate buffer. The final dNTP concentration was 3 nM, the oligonucleotide concentration 3.35 pM. PCR reactions were performed with the Thermal-Cycler PTC-200 (GMI, Minnesota, USA).

The amplified products were purified by gel extraction from a 1.5% agarose gel with the Gel-Extraction-Kit (Qiagen, Hilden, Germany) according to the manufacturer's specifications and enriched with 1 mM guanosine to stabilise the DNA.

### Subcloning

After purification 65 ng of each amplified product was cloned into a pDrive-cloning vector and transformed into *E. coli *cells (Qiagen competent cells, Hilden, Germany) according to the manufacturer's specifications (Subcloning-Kit, Qiagen, Hilden, Germany). A volume of 100 μl of the bacterial suspension was transferred to an LB-agar plate containing 100 μg/ml ampicillin, 80 μg/ml XGal and 50 μM IPTG for blue/white screening. A single agar plate was used for each oligonucleotide combination or PCR-product, respectively. Accordingly 18 agar plates were inoculated. The plates were incubated for 18 h at 37°C and finally for 3 h at 4°C.

Twenty colonies per plate were selected (a total of 360 colonies) and transferred to 24-well plates (Sarstedt, Nümbrecht, Germany) whose wells were filled with LB-agar enriched with 100 μg/ml ampicillin. A colony PCR was performed with the oligonucleotides T7 promoter-oligo in combination with the SP6 promoter-oligo (Table 1) to confirm the presence of the inserted product. Therefore, each colony was used as a template and the reaction mixtures were incubated at 94°C for 3 min, followed by 35 cycles at 50°C for 50 s, 68°C for 50 s, and 94°C for 25 s. A final extension step was performed at 68°C for 10 min. The Hot-Start-polymerase (Eppendorf, Hamburg, Germany) was used (1.5 U/reaction) with the appropriate buffer. The oligonucleotide concentration was 3.35 pM, the dNTP concentration 3 nM.

### Sequencing of individual clones

Two hundred and sixty-nine positive clones were selected for sequencing. The sequencing reactions were performed at the Institute for Services in Molecular Biology and Biochemistry (DLMBC) in Berlin, Germany. The colonies embedded in the 24-well plates were directly sent to the DLMBC. Sequencing reactions were performed with the oligonucleotides T7 promoter-oligo and SP6 promoter-oligo (Table 1), products were sequenced forward and reverse.

### Evaluation of the cDNA-sequences

The obtained sequences were evaluated by using the software programs BioEdit 7.0.4.1 ([31]; http://www.mbio.ncsu.edu/BioEdit/BioEdit.html) and Sequencher 4.8 (Gene Codes Corporation, Ann Arbor, USA; http://www.genecodes.com).

Matching forward and reverse sequences were compared (BioEdit 7.0.4.1). Sequences with discrepancies based on the sequencing reaction were excluded. Sequences with an identity exceeding 99% were pooled into one contig (Sequencher 4.8) and the consensus sequence was created. Ambiguities at certain base positions (nucleotide polymorphisms) can be due to an amplification error of the polymerase, a sequencing error, possible gene duplications, or an SNP. The most frequent base at a position was chosen for the consensus sequence. Some of the sequences could not be integrated into a contig and were consequently treated separately.

To prove this method of evaluation, the sequences were processed in an alternative way: DNA-sequences with an identity of 100% were pooled into contigs (Sequencher 4.8) and the consensus sequences were created. The contigs and discrete sequences were translated. The resulting amino acid sequences were aligned and sequences with an identity of more than 99% were again pooled into contigs (Sequencher 4.8). In the case of varieties at one position, the most frequent amino acid at this position was chosen for the consensus sequence but according to the following premises: First, an amino acid was exchanged only if solely this amino acid at the specific position inside the contig was ambiguous. If two or more amino acids were ambiguous at one specific position, a new contig was created. Secondly, inside an amino acid sequence only one amino acid exchange was allowed, otherwise the sequence was processed as an individual peptide. Finally, the amino acid sequences obtained by using the first method were aligned with the sequences obtained from the second one.

### Comparison of the transcripts with genomic sequences

To confirm the transcripts a genome-screening was performed in the equine database with the cDNA-sequences as templates to recover the genes associated with the transtripts. To determine the genomic positions of the obtained transcripts we performed a database search using BLAT http://genome.ucsc.edu/cgi-bin/hgBlat with the following settings: genome, Horse; assembly, September 2007; query type, DNA.

The cDNA sequences from the ATG-start codon to the end of the 3'-UTR of the transcripts were used. If the identity of a transcript and a genomic region exceeded a value of 99%, they were defined as matching. We compared not only the whole transcript with the genomic sequence but also the individual exons. We excluded false-positive matches of whole transcripts based on a high identity of a single exon (>99.5%) but low identity (<99%) of the other exon. For example: With *DEFA3 *we found a matching genomic sequence showing an identity of 99.1%, whereas exon 1 shows an identity of 100% and exon 2 only 97.5% (this is equivalent to a difference of 3 bp). Accordingly, a complete transcript was only matched to a gene if both the first and the second exon showed an identity exceeding 99%.

## Results

### Genomic α-defensin sequences

Twenty-nine different nucleotide sequences resembling α-defensin genes were obtained from the wgs-database of the horse by using the DEFA5L- and DEFA1 sequences as search matrices. The accession numbers and positions of the genomic sequences are shown in Table 2.

**Table 2:** Whole genome α-defensin like sequences (wgs) of the horse.

**Acc. no**.	Position	Strand	Chromosome and Position	
AAWR01019889	174567-175373	+	27	33528746-33529552
AAWR01019889	184465-185420	+	27	33538643-33539598
AAWR01019889	200883-201833	+	27	33555053-33556053
AAWR01019889	210924-211864	+	27	33565089-33566029
AAWR01019889	220569-221379	+	27	33574734-33575544
AAWR01019889	231156-232116	+	27	33585321-33586281
AAWR01019889	242018-242966	+	27	33672516-33673464
AAWR01019889	250299-257468	+	27	33604485-33611671
AAWR01019891	000001-000677^a^	+	27	33620983-33621659
AAWR01019891	004195-005146	+	27	33625177-33626128
AAWR01019891	014325-015118	+	27	33635311-33636104
AAWR01019891	023449-024204^b^	+	27	33644436-33645191
AAWR01019891	032877-033688	+	27	33653858-33654669
AAWR01019891	040655-041615	+	27	33661636-33662596
AAWR01019892	000917-001202^b^	+	27	33680242-33680527
AAWR01019892	008326-009116	+	27	33687651-33688442
AAWR01019892	018850-019804	+	27	33698171-33699123
AAWR01019892	029831-030772	+	27	33709150-33710090
AAWR01037147	002817-003615	-	Un^f^	46286046-46286850^g^
			Un	46298545-46299353^g^
AAWR01037147	012991-013946	-	Un	46296219-46297174
AAWR01037147	015317-016125	-	Un	46298545-46299353
AAWR01037147	020479-020879^b^	-	Un	46303706-46304106
AAWR01037147	025848-026018^c^	-	Un	46309075-46309245
AAWR01037147	028029-028833	-	Un	46311256-46312060
AAWR01037946	002841-003485^d^	-	Un	46326945-46327589
AAWR01037947	002474-003275	-	Un	46335947-46336748
AAWR01038861	002768-003577	-	Un	46286046-46286850^g^
			Un	46298545-46299353^g^
AAWR01039498	006542-006682^e^	-	Un	85437360-85437500
AAWR01043576	002486-003610^b^	-	27	33679407-33680531

The sequences (based on assembly EquCab1.0) are given from start codon to 3'-end of putative transcript. The chromosomal position is based on assembly EquCab2.0.

^a ^Exon 1 truncated at 5'-end. ^b ^3'-end of intron, exon 2. ^c ^Mutated exon 1. ^d ^Exon 1, 5'-end of intron. ^e ^3'-end of exon 2. ^f ^unknown chromosomal assignment. ^g ^Mismatches, two putative positions or not present in EquCab2.0.

### Repertoire of the α-defensin transcripts

The alignment of cDNA-sequences with an identity of more than 99% resulted in a total of 37 different cDNA sequences. After translation of the cDNA sequences 35 different amino acid sequences could be determined (first evaluation method).

In the case of the second evaluation method a total of 150 different cDNA sequences resulted if all cDNA-sequences with an identity of 100% were aligned. After translation and alignment of the amino acid sequences with an identity of 100%, 98 different peptides were found. The amino acid sequences were finally corrected as described in the Material section and as a result 34 different peptide sequences were obtained.

**Figure 1:**
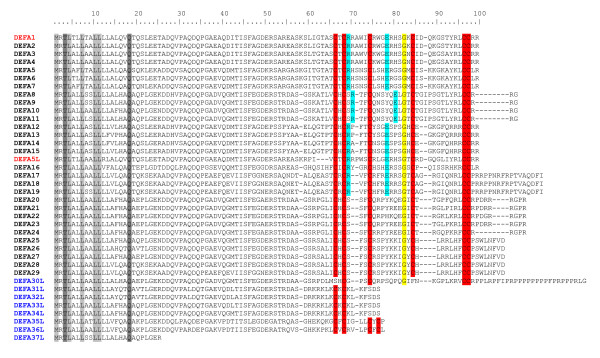
**Alignment of 38 different α-defensin amino acid sequences**. Fig. 1 represents the conserved amino acid residues and distinguishes potentially active peptides from pseudogenes. Conserved amino acid residues in the signal peptides present in all sequences were highlighted in grey. Conserved cysteine residues were highlighted in red, conserved arginine and glutamic acid residues, necessary for the intramolecular salt bridge were highlighted in blue, the conserved glycine residue, necessary for correct folding is highlighted in yellow. The previously known α-defensins DEFA1 and DEFA5L are written in red, defensin-like peptides (pseudogenes) in blue.

**Figure 2:**
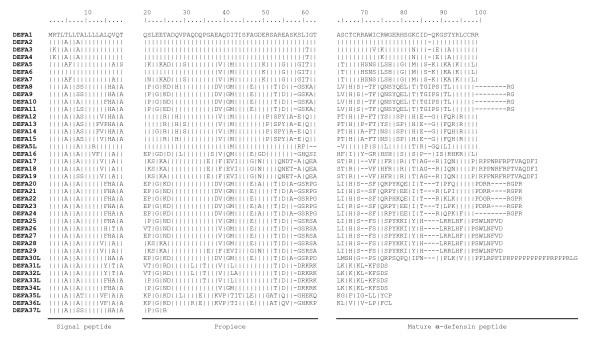
**Divergences between the equine intestinal α-defensins**. Fig. 2 emphasises the clustering of the peptides. The predicted signal sequence, the propiece, and the mature peptide were shown separately. DEFA1 was used as a prototype and the amino acids of the other peptides identify only those residues that differ from the DEFA1 sequence. Identical residues are represented by a vertical line.

The 35 amino acid sequences of the first evaluation method were aligned with the 34 sequences obtained with the second evaluation method and peptides with an identity of 100% were combined. An alignment of the resulting 38 different peptide sequences is shown in Fig. 1 and Fig. 2. Table 3 shows the number of obtained clones along with the cDNA sequences corresponding to the amino acid sequences shown in Fig. 1 and Fig. 2 and the accession numbers of the equine α-defensin transcripts.

**Table 3:** Number of obtained clones (No.) and accession numbers (Acc. no.) of the equine α-defensin transcripts.

Transcript	**No**.	**Acc. No**.
DEFA1	3	[GenBank:EF379126]
DEFA2	1	[GenBank:GQ259765]
DEFA3	1	[GenBank:GQ259766]
DEFA4	19	[GenBank:GQ259767]
DEFA5	38	[GenBank:GQ259768]
DEFA6	1	[GenBank:GQ259769]
DEFA7	2	[GenBank:GQ259770]
DEFA8	9	[GenBank:GQ259771]
DEFA9	16	[GenBank:GQ259772]
DEFA10	2	[GenBank:GQ259773]
DEFA11	1	[GenBank:GQ259774]
DEFA12	17	[GenBank:GQ259775]
DEFA13	1	[GenBank:GQ259776]
DEFA14	3	[GenBank:GQ259777]
DEFA15	1	[GenBank:GQ259778]
DEFA16	2	[GenBank:GQ259779]
DEFA17	29	[GenBank:GQ259780]
DEFA18	4	[GenBank:GQ259781]
DEFA19	1	[GenBank:GQ259782]
DEFA20	4	[GenBank:GQ259783]
DEFA21	1	[GenBank:GQ259784]
DEFA22	2	[GenBank:GQ259785]
DEFA23	4	[GenBank:GQ259786]
DEFA24	2	[GenBank:GQ259787]
DEFA25	39	[GenBank:GQ259788]
DEFA26	1	[GenBank:GQ259789]
DEFA27	1	[GenBank:GQ259790]
DEFA28	1	[GenBank:GQ259791]
DEFA29	2	[GenBank:GQ259792]
DEFA30L	2	[GenBank:GQ259793]
DEFA31L	27	[GenBank:GQ259794]
DEFA32L	1	[GenBank:GQ259795]
DEFA33L	1	[GenBank:GQ259796]
DEFA34L	1	[GenBank:GQ259797]
DEFA35L	3	[GenBank:GQ259798]
DEFA36L	4	[GenBank:GQ259799]
DEFA37L	1	[GenBank:GQ259800]
DEFA5L	2	[GenBank:GQ259801]

All sequences are in a range of 25 to 124 amino acid residues but not all sequences seem to be potentially functional α-defensins. In some sequences typical conserved α-defensin motives are missing in the mature peptide segment. These include the cysteine residues, necessary for the typical defensin disulfide-bond connectivity; arginine and glutamate residues, forming a conserved salt bridge [32]; and a highly conserved glycine residue, essential for correct folding [33]. The peptides DEFA20 to DEFA29 contain the six cysteine residues at position 66, 68, 74, 84, 96, and 97 and the glycine residue at position 82 but they have lost the intramolecular salt bridge. DEFA30L is also missing the salt bridge and additionally the two cysteine residues at positions 66 and 84. The peptides DEFA31L to DEFA37L exhibit a premature stop codon, the translation stopped at position 77 (DEFA31L to DEFA34L), 78 (DEFA35L, DEFA36L) or at position 26 visible in the appropriate cDNA sequence. DEFA30L to DEFA37L appear to be pseudogenes. In the case of DEFA20 to DEFA29 it has to be proved whether the intramolecular salt bridge leads to a loss of the biological function. DEFA1 to DEFA19 and DEFA5L show all conserved amino acid motives typical for α-defensins. It can be assumed that at least these 20 peptides are functional molecules and have the potential to exhibit antimicrobial properties as already shown for DEFA1 [18,29].

### Comparison of the transcripts and genomic sequences

To confirm the obtained transcripts the matching genomic sequences were determined, as shown in Table 4. Ten transcripts lacked matching genomic sequences, for further ten transcripts the first exon cannot be found in the genomic database and for eight transcripts the second exon cannot be found. On the other hand, for 14 α-defensin genes apparently present in the genome (Table 2) no appropriate transcript could be verified.

**Table 4:** Genomic sequences compared with obtained α-defensin transcripts.

Chromosome/Strand/Position	Length	Matching transcripts
27 + 33528746-33529555	810	DEFA31L 100% DEFA32L 99.6%
		Exon1 99.5%
		Exon2 99.6%
27 + 33529309-33529547	239	DEFA33L, Exon2 99.6% DEFA34L, Exon2 100%
27 + 33538643-33539595	953	DEFA5L 99.6%
		Exon1 99.5%
		Exon2 99.6%
27 + 33555053-33555227	175	DEFA3, Exon1 100% DEFA4, Exon1 99.5%
27 + 33565089-33566021	933	DEFA12 99.8%
		Exon1 100%
		Exon2 99.6%
		DEFA13, Exon2 100%
		DEFA15, Exon1 100%
27 + 33585321-33586281	953	DEFA7 99.8%
		Exon1 100%
		Exon2 99.6%
		DEFA6, Exon2 100%
27 + 33621410-33621658	249	DEFA8, Exon2 100% DEFA9, Exon2 99.2% DEFA10, Exon2 99.2%
27 + 33654421-33654662	242	DEFA35L, Exon2 99.6%
27 + 33662350-33662588	236	DEFA5, Exon2 100% DEFA7, Exon2 100%
27 + 33709150-33710093	944	DEFA17 99.6%
		Exon1 100%
		Exon2 99.2%
		DEFA18 99.8%
		Exon1 100%
		Exon2 100%
		DEFA19, Exon2 99.2
		DEFA29, Exon1 100
Un - 46286679-46286850	172	DEFA21, Exon1 100% DEFA22, Exon1 99.5
Un - 46298552-46299353	802	DEFA23 100% DEFA20, Exon1 99.5%
Un - 46299182-46299353	172	DEFA24, Exon1 99.5%
Un - 46311263-46312060	798	DEFA16 99.8%
		Exon1 99.5%
		Exon2 100
Un - 46335954-46336748	795	DEFA36L 100%

Identities in % were determined by BLAT-search on horse assembly (EquCab2.0).

**Figure 3:**
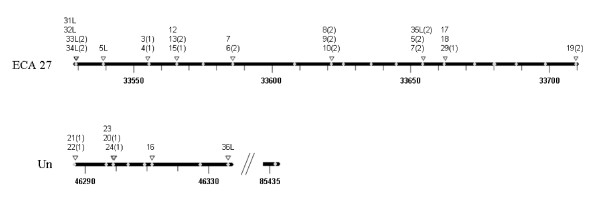
**Positions of α-defensin transcripts and genomic gene sequences based on assembly EquCab2.0**. A genomic region of approximately 183 kb of chromosome 27 (top) and a second region of approximately 50 kb of an unspecified segment (bottom) are shown. Positions of genomic gene sequences are depicted as grey dots, positions of transcripts as triangles. Complete transcripts are identified by numbers according to the peptides' name. If only single exons are located at a specific position, this is indicated by the specification of the accordant exon written in parenthesis after the peptides' name. The scale indicates the kb in the chromosomal region.

The positions of the transcripts and genomic sequences (Table 2) which are located in a genomic region of 181,347 bp on ECA27 and another region of 50,069 bp on an unspecified genomic fragment (Un), with one exception ([GenBank:AAWR01039498]), are shown in Fig. 3.

## Discussion

In this study we demonstrated the expression of a large repertoire of different α-defensin transcripts in the small intestine of a single horse. Consequently, this study is not to be considered representative for α-defensins in horses generally and conclusions regarding differences between races or states of health cannot be drawn. However, the extensive quantity of at least 38 detected α-defensin transcripts (30 probably functional peptides, DEFA1 to DEFA29 including DEFA5L, and eight obvious pseudogenes, DEFA30L to DEFA37L) in the intestinal tissue has rarely been observed before in other organisms and may indicate an important role of these AMPs in horses. The functional context of this high number of different transcripts is yet unclear.

In the human Paneth cells two different α-defensins are synthesised (DEFA 5 and DEFA6; [34]) in addition to the neutrophil human α-defensin peptides 1-4, also known as HNP 1-4. In the intestine of the rhesus macaque four different peptides are observed, RED-1 to RED-4 [35]. The only known organism with a numerically related repertoire of enteric α-defensins is the mouse. At least 23 different peptides are known, named cryptdins and cryptdin-related sequences [36-38]. They were not only found in the small intestine but also in the colon, the cecum and the rectum. The antimicrobial activities of some of these cryptdins are not known. In mice three different observations highlight possible advantages to express a high amount of different defensin peptides and indicate their biological relevance.

First, the peptides can exhibit different specificities against microorganisms. The cryptdins 1 to 6 were analysed regarding their antimicrobial activity against different bacteria. The killing potency against the targets, particularly *E. coli *and *Salmonella typhimurium*, showed variabilities [37].

Secondly, the expression level of the cryptdin genes along the intestinal tract showed large variations. Karlsson et al. [36] observed a differential gene expression of cryptdins in the duodenum, jejunum and ileum. They concluded that the varying cryptdin synthesis in different tracts of the intestine may play an important role in the local regulation of bacterial colonisation in addition to the role in protection against infection. In having analysed only a single intestinal location, similar conclusions cannot be drawn from our data. However, different cloning frequencies of the transcripts found in this study may indicate different mRNA-expression levels at least at this location. The number of cDNA-fragments coding for a specific peptide varies from one (e.g. DEFA2, DEFA3) to 39 (DEFA25) in randomly selected clones. Although 38 different α-defensin transcripts were observed in this study, only seven cDNAs were cloned frequently (DEFA4, 5, 8, 9, 12, 17, 31L, Table 3), whereas in all other cases the number of obtained clones varies between one and four. Since only a single set of amplification products was cloned and variants due to error-prone PCR are possible, these data are less representative. However, the same situation exists in the mouse, where over 30 α-defensin genes were included in the latest assemblies and over 20 different cDNAs were reported, but only six cDNAs have been cloned at high frequencies. It should be taken into account that despite the apparent diversity, it might be unlikely that more than 7-8 peptides accumulate to considerable levels in the small intestine of the horse. Furthermore, it is not known whether and to what extent the gene expression is inducible.

Thirdly, the gene expression of different mice cryptdins shows circadian variances [39]. The expression level varies about 100% between simulated dark and light phases, primary between cryptdin 1 and 4. An interrelation with the time of ingestion was assumed. Moreover, the gene expression level of cryptdin 2 and 5 changes about two or three orders of magnitude in the first days after birth whereas the gene expression of cryptdin 1, 3, and 6 increases at lower pace [40]. Probably the differential gene expression protects the organism during the first adaptation to a new environment.

**Figure 4:**
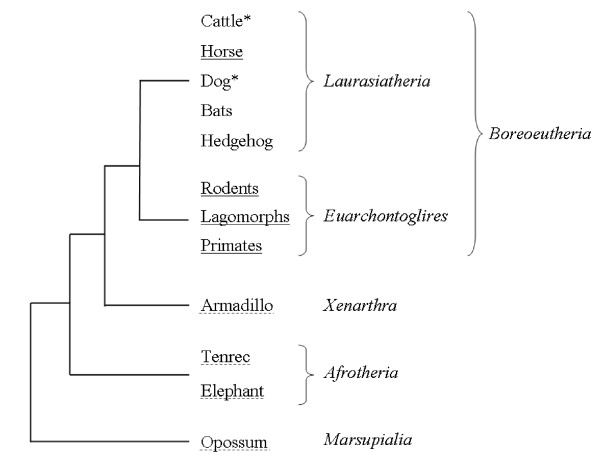
**Phylogeny of the mammals based on the Bayesian phylogenetic tree according to Murphy et al**. [41]. In underlined species α-defensin genes and transcripts are known. In species underlined with a dashed line, α-defensin genes in the genome are only known by *in silico *approaches [20]. The asterisked species have no α-defensin genes.

Alpha-defensin transcripts were formerly only known in primates, glires and horses. In recent studies the existence of α-defensin genes was observed in the genomes of the opossum [19], the elephant and the hedgehog tenrec [20]. According to the Bayesian phylogenetic tree of mammals [41], the horse is classified into the group of the *Laurasiatheria *as well as cattle, dog, bat and hedgehog (Fig. 4). Nothing is known about the existence of α-defensins in bat and hedgehog, and no α-defensin gene was found in cattle [21] and dog [22]. Whereas α-defensin genes exist in basal mammals like opossum, elephant and hedgehog tenrec and in the group of *Euarchontoglires*, the *Equidae *are the only known family expressing α-defensin genes within the group of *Laurasiatheria*. In future studies it will be necessary to clarify why cattle and dog presumably lost their complete set of α-defensin genes while the horse increased the gene number extensively. Additionally, the presence or absence of α-defensins in the closest relatives of the horse like tapir and rhinoceros (which form together with the horse the group of *Perissodactyla*) has to be analysed. This may lead to new aspects in the development, reorganisation and separation of defensin genes. It is very unlikely that α-defensins evolved independently within the *Equidae*, indicated by the high analogy between the amino acid sequences of the horse compared with known α-defensins from primates and glires [18]. It is assumed that α-defensins evolved from one or two ancestral genes by gene-duplication [22,42]. According to the observation that platypus being the most basal mammal that already has been sequenced has four α-defensin genes [43], one can hypothesise that α-defensins may have been lost independently in different clades during the divergence of the phylogenetic tree.

The genomic positions of the transcripts or of single exons, respectively, as shown in Fig. 3 indicate a false assembly of EquCab2.0 or unidentified gaps in the genomic sequence. In some cases only one exon of a transcript was found with an identity of 100% in the genome whereas an appropriate second exon was missing (*DEFA6*, *DEFA8*, *DEFA13*). In other cases the same genomic exons were found in different transcripts (*DEFA6 *and *DEFA7 *(Exon 2), *DEFA12 *and *DEFA13 *(Exon 2), *DEFA12 *and *DEFA15 *(Exon 1)). The second exons of the *DEFA6 *and *DEFA7 *transcripts were located at the same genomic position whereas only the first exon of *DEFA7 *was identified at an appropriate distance of approximately 700 bp upstream. The first exon of *DEFA6 *could not be detected in the genome indicating a gap. In addition to assembly errors, comprehensive gene reduplication and mutations of α-defensins and/or alternative splicing might be possible explanations, although the latter was never shown for α-defensins before. Additionally it is possible that transcripts with missing genomic sequences in the assembly EquCab2.0 could be also due to copy number variations (that have e.g. been reported for human α-defensins; [44]). The animal used in our investigations might have possessed additional copies compared with the horse used for establishing the reference genome. However, Fig. 3 shows a typical clustering of the equine α-defensins with average distances of approximately 10 kb between single genes. No transcripts were found in the small intestine for some α-defensin genes annotated in the genome of the horse or identified by BLAST-searches. It is possible that these genes are expressed in other tissues and cells, e.g. bone marrow, or are expressed at very low levels in the small intestine. In other species that have enteric α-defensins, they are the products of Paneth cells. Their number varies in different sections of the small intestine (duodenum, jejunum and ileum) and certain α-defensins were only found in the ileum [36]. In our studies we most probably used tissue samples of the jejunum. Consequently, our results do not allow a differentiation between the single sections of the small intestine and it is possible that some transcripts may be additionally found exclusively in the duodenum and/or ileum.

The primary structure of mature α-defensin shows highly conserved residues, which are indispensable for the structural stability and the full function of the peptides. Among them are six invariant cysteine residues, necessary for the typical α-defensin intramolecular disulfide-bond connectivity (Cys_1_-Cys_6_, Cys_2_-Cys_4 _and Cys_3_-Cys_5_), two charged amino acid residues, Arg_5_, and Glu_13_, forming a conserved salt bridge [32], and Gly_17_, which constitutes a structural motif which is essential for correct folding [33]. By analysing the new equine α-defensins considering that these prerequisites must be fulfilled for an active α-defensin, it became evident that 20 of the 38 peptides (including the known DEFA1) may be active α-defensins with an antimicrobial potential. DEFA1 to DEFA19 and the DEFA5L transcript show the six conserved cysteine residues, the arginine and the glutamic acid residues necessary for the intramolecular salt bridge, and the highly conserved glycine residue necessary for correct folding. The α-defensins DEFA20 to DEFA29 exhibit also the cysteine and glycine residues but they have lost the intramolecular salt bridge formed by Arg_5 _and Glu_13_. Nonetheless, they should be considered functional because this bridge is not required for correct pro-α-defensin folding [32]. In contrast, the peptides DEFA30L to DEFA37L appear to be pseudogenes. Either some cysteine residues were absent (DEFA30L) or the transcription stops before completion because of a premature stop codon visible in the appropriate cDNA sequence. It is not known whether these molecules may have adopted new functions or are involved in regulatory systems. Predictions (e.g. as non-coding RNAs) at this point would be highly speculative. Interestingly, the premature stop codon of DEFA35L and DEFA36L at position 78 is at the same position that gives rise to θ-defensins in non-human primates [45].

Typically, α-defensins have an anionic propeptide and a cationic mature peptide, and the charges are counterbalanced. Taking all intestinal α-defensins of the horse into account that can produce a mature peptide (DEFA1-DEFA29, DEFA5L) they follow the pattern of anionic propiece and cationic mature peptide, but only six of them are counterbalanced (DEFA8, 12, 13, 15, 21, 27). Several of the equine α-defensins (DEFA18-20, DEFA30L) have proline-rich C-terminal extensions resembling certain proline-rich antimicrobial peptides of cattle, named cathelicidins (CATHL2 and CATHL3) and others in sheep and goats [46]. Also DEFA30L exhibits a proline-rich C-terminal region and four cystein residues, typical for cathelicidins, but the spacing of the cysteins is different. Other equine α-defensin peptides have a C-terminal glycine residue (DEFA8 to DEFA11). Many cathelicidin precursors also have a C-terminal glycine, which allows the peptide's C-terminus to be amidated. To our knowledge no defensins from other species are amidated. The only other α-defensin containing a C-terminal glycine is DEFA3 of platypus (*Ornithorhynchus anatinus*, acc. Nr. P0C8A3, Swissprot: http://www.expasy.ch/sprot).

Different infectious diseases may appear if the expression level or intracellular processing of Paneth cell α-defensins is abnormal. Wehkamp et al. [26] discovered a reduced Paneth cell α-defensin synthesis in ileal Crohn's disease (Morbus Crohn), a chronic disease of the intestine, by using real-time PCR and immunohistochemical methods. An enhanced expression of epithelial α-defensin genes in colonic inflammations was reported by Wehkamp et al. [47]. Ferguson et al. [27] found that single nucleotide polymorphisms in the human Paneth cell α-defensin DEFA5 may confer susceptibility to inflammatory bowel disease. Inflammatory bowel diseases have also been described in horses, for example the duodenitis/proximal jejunitis syndrome which is characterised by catarrhal enteritis with mucosal hyperaemia or necrosis or colitis, which causes intramural oedema and haemorrhagic inflammation of the large intestine and is often fatal. Bacteria have been implicated as etiological agents of both diseases [48,49]. The high quantity of equine intestinal α-defensins and the considerable biological effect of enteric DEFA1 against microorganisms emphasises the importance of equine intestinal defensins in protection of the horse against infections of the intestinal tract.

## Conclusions

The large quantity of at least 38 equine α-defensin transcripts in the intestinal tissue has rarely been observed before in other species. In previous studies we could show the comprehensive antimicrobial activities of one of these molecules. The biological context of expressing such a high number of different α-defensins in the intestine remains unclear. It is likely that the peptides exhibit different specificities against microorganisms, or different requirements for antimicrobial peptides along the intestinal tract may lead to spatial expression patterns. Also temporal expression variations may be possible, e.g. a regulation of α-defensins by circadian rhythms. However, the high quantity of equine intestinal α-defensins emphasises their importance in the health of the horse's gut. Our study provides the basis for further gene-expression studies in the intestinal tract of the horse or biological analyses of further equine α-defensins to understand the importance of defensins in the enteric innate immunity. Moreover, the repertoire of equine α-defensins make the horse an excellent species to study the biological properties of α-defensins regarding animal health. From other species it is known that deficiencies or dysregulations of Paneth cell α-defensins lead to enteric infectious diseases.

A comparison of the genomic sequences with the obtained transcripts indicates a false assembly of the sequences in the genomic databases of the horse or unidentified gaps. Large gene duplication events and mutation of α-defensins may have occurred in the horse.

## List of abbreviations used

acc. No: accession number; BLAST: basic local alignment and search tool; BLAT: BLAST like alignment tool; DEFA: defensin α; DEFAL: defensin-α like; EST: expressed sequence tag; HNP: human neutrophil peptide; RED: rhesus macaque enteric defensin; RT: reverse transcriptase; U: unit; wgs: whole genome shotgun sequences

## Authors' contributions

OB prepared the RNA samples, performed the sub-cloning experiments and did the major part of the analysis of the transcripts, participated in the design of the study and did the mayor part of drafting the manuscript. SP did the major part of the genomic database analysis and participated in the comparative studies of genomic sequences and transcripts. SP participated in the drafting of the manuscript. JT participated in the design and the data analysis of the study and the interpretation of results. GT conceived and coordinated the study and refined the manuscript. All authors were involved in a critical revising and approved the final manuscript.
